# The experiences of therapists providing cognitive behavioral therapy (CBT) for dissociative seizures in the CODES randomized controlled trial: A qualitative study

**DOI:** 10.1016/j.yebeh.2020.106943

**Published:** 2020-04

**Authors:** Matthew Wilkinson, Elana Day, James Purnell, Izabela Pilecka, Iain Perdue, Joanna Murray, Edyta Monika Hunter, Laura H. Goldstein

**Affiliations:** a Canterbury Christ Church University, Salomons Institute for Applied Psychology, Tunbridge Wells, UK; b South London and Maudsley NHS Foundation Trust, London, UK; c Institute of Psychiatry, Psychology and Neuroscience, King's College London, London, UK

**Keywords:** Dissociative seizures, Cognitive behavioral therapy, Randomized controlled trial, Qualitative

## Abstract

**Objectives:**

Little is known about the experiences of therapists delivering psychotherapy for patients with dissociative seizures (DS), a complex disorder associated with a range of comorbid psychosocial and mental health difficulties. This study set out to explore therapists' experiences of delivering DS-specific, manualized cognitive behavioral therapy (CBT) to adults with DS within the context of a randomized control trial.

**Methods:**

Interviews were conducted with 12 therapists involved in the COgnitive behavioral therapy vs standardized medical care for adults with Dissociative non-Epileptic Seizures (CODES) trial and were analyzed using thematic framework analysis (TFA).

**Results:**

Six main themes emerged, namely 1) aspects of the intervention that were favored, while others were not always considered applicable; 2) multiple and complex difficulties faced by patients; 3) working effectively within the protocol; 4) limitations of the protocol; 5) significance of formulation; and 6) quality of standardized medical care (SMC) and difficulties of diagnosis delivery. These addressed valued aspects of the intervention, complexities of the patient group, and experiences working within a structured treatment protocol. Family involvement and psychoeducation were highlighted as important components; the applicability of graded exposure techniques, however, was restricted by patients' apparent emotional avoidance. The structure provided by the treatment protocol was valued, but flexibility was important to individualize treatment in complex cases. A comprehensive formulation was fundamental to this. The initial diagnostic explanation provided by neurologists and psychiatrists was generally considered beneficial, with patients often perceived to enter therapy with a better understanding of their condition.

**Conclusions:**

This study demonstrated that the DS-specific CBT intervention met with general approval from therapists who also highlighted some practical challenges. Because of the nature of the condition, the need for experience of working with complex patients should be considered when applying the intervention to individual cases. Setting the CBT intervention in the context of a structured care pathway involving neurology and psychiatry may facilitate the therapeutic process.

## Highlights

• Therapists can use DS-specific CBT with patients with DS, despite challenges.
• Clinical experience and understanding patients' complexity are important.
• Clinical formulation is an important aspect of this DS-specific CBT.
• Flexibility in ordering treatment components of this manualized therapy is important.

## 1 Introduction

Dissociative seizures (DS) are the most common functional neurological disorder encountered by neurologists [1,2]. They involve paroxysmal episodes that may superficially resemble epileptic seizures or syncope but which might have distinguishing features such as longer duration of symptoms, eye closure during episodes, and memory recall [3], although this is not always the case. Qualitative research has highlighted the potentially debilitating and isolative experiences of living with DS [4,5].

Psychological models have emerged which help to account for the symptoms and phenomenology of DS. Early concepts suggested that episodes arise as a result of the dissociation of psychological processes in response to trauma [6], or as a ‘conversion’ of traumatic emotional responses into physiological responses [7]. More recent approaches have adopted the fear-avoidance model [[8], [9], [10]]. Further research suggesting that trauma is a common but not necessary factor in developing DS, has led to the development of additional psychological models, for example the ‘Integrative Cognitive Model’ [11]; other models include the ‘panic-without-panic’ model of DS [12].

Psychotherapy is often considered to be the treatment of choice for DS [13] despite the absence of good quality research evidence to date [14]. Of note here has been the application of cognitive behavioral therapy (CBT) to treating DS. Cognitive behavioral therapy is a talking therapy predicated on the concept that a person's thoughts, feelings, physical sensations, and actions are interrelated so that, for example, thoughts and beliefs can affect feelings and behavior. Of relevance here, studies have included two CBT-based approaches in DS management: ‘CBT-informed psychotherapy’ (CBT-ip; [15]) and an approach based on the fear-avoidance model [8,10]. While DS frequency is usually the primary outcome in studies, other outcomes such as anxiety, depression, and other measures of psychosocial function are often included. For example, LaFrance et al. [15] conducted a small pilot randomized clinical trial (RCT) and showed a statistically significant reduction in seizures and some secondary outcomes with CBT-ip without and with a flexible sertraline dose. However, the study was insufficiently powered to allow comparisons between treatment arms.

The fear-avoidance model [8,10] is of relevance to patients with DS since despite an absence of subjective anxiety as part of their seizures, many individuals with this condition exhibit high levels of avoidant behavior and coping styles [12,[16], [17], [18]]. The treatment for patients with DS in subsequent studies including the COgnitive behavioral therapy vs standardized medical care for adults with Dissociative non-Epileptic Seizures (CODES) study was initially based partly on the fear-avoidance model [9]. Chalder's [9] single case study reported seizure cessation following CBT. Additionally, a small open label study was conducted mirroring the 12-session CBT intervention, resulting in a reduction in seizure frequency [19] and other beneficial outcomes. This led to a pilot randomized controlled trial (RCT) [20] using DS-specific CBT alongside standard medical (neuropsychiatric) care which showed a statistically significant reduction in seizure frequency compared with standard medical care alone at the end of treatment.

Following the pilot RCT, Goldstein and colleagues have conducted an adequately powered RCT called the ‘CODES trial’ [21]. This was conducted within the National Health Service (NHS), and trial patients received clinical care from neurology and liaison/neuropsychiatry services even where these may not typically have been available outside of the trial [22]. Cognitive behavioral therapy was provided for those patients randomized to receive it. In this study, 368 patients with DS were randomized to receive 12 sessions of CBT (plus a booster session) in addition to standardized medical care (SMC) or SMC alone. Standardized medical care doctors (neurologists and psychiatrists) were provided with written supporting materials. Standardized medical care incorporated a recommended manner in which to deliver the diagnosis, and the provision of additional resources such as information booklets (see http://www.codestrial.org/information-booklets/4579871164). It also comprised follow-up with guidance regarding the possible content of follow-up sessions. These sessions were meant to be supportive, could include pharmacotherapy for comorbidities and general review, but the clinicians were asked to refrain from using CBT techniques [21]. The DS-specific CBT approach was based on previous studies [9,19,20]. The DS-specific CBT treatment incorporated engagement and psychoeducation (predominantly sessions 1–2), techniques for seizure management (session 2 onwards), reducing fear avoidance and use of exposure (introduced in session 2), identification of and challenging seizure-related cognitions (and where relevant, addressing trauma; session 4 onwards), and relapse prevention (session 10 onwards). Content invariably straddled several sessions, and it was acknowledged that individual presentations might require some reordering of components. Therapists of CODES CBT also provided patients with a handbook of chapters on a range of topics (see Supplementary Material 1) to supplement the treatment sessions. Therapists received specific training for the study, a session-by-session therapy manual as guidance, and telephone-based group (and occasionally individual) supervision for CODES-specific issues with one of the three senior CBT therapists (who were very experienced in delivering this model, having a median of 10 years' (range: 10–30 years) experience working with this therapeutic model) every four to six weeks throughout the treatment phase of the trial.

Little is known about therapists' experiences delivering treatment to patients with DS (particularly those involving CBT) [23]. The most comparable interview-based study used a grounded theory approach to examine psychotherapists' understanding of this disorder by focusing on successfully delivered, longer-term interventions only [24]. The aim of the present study was to investigate the experiences of the CBT therapists involved in the CODES RCT. This aspect of the research was intended to supplement the quantitative outcomes of the RCT and other related qualitative studies (experiences of the psychiatrists and participants in the CODES trial) to provide insight into factors that might affect psychological intervention delivery for patients with DS.

The main objectives of the present study were to investigate the following:

• how therapists dealt with the delivery of a structured, novel intervention for DS,
• which aspects therapists found particularly useful or challenging,
• whether the complex difficulties faced by this patient group (e.g., previous trauma, general anxiety) [25] presented a challenge in the application of a manualized, structured treatment, and
• therapists' opinions of SMC, given the significance of effective diagnosis and multidisciplinary working when working with functional neurological conditions [2].

## 2 Methods

### 2.1 Participants

Participants were NHS clinicians delivering structured CBT to individuals with DS within the CODES RCT. The trial received ethical approval from the London – Camberwell St Giles Research Ethics Committee and the Health Research Authority (REC reference: 13/LO/1595).

As the CODES RCT involved 39 therapists based within 18 NHS trusts throughout the UK, it was not logistically feasible to conduct and analyze interviews with all therapists. Purposive sampling was employed to identify a subgroup of clinicians that exhibited diversity in terms of professional background, years of experience working with DS, level of experience using this model within the trial, and geographical location. Existing research provides evidence that professional background and level of experience are associated with divergent views regarding the condition [26,27]. Given evidence of regional variation in treatment delivery [22], it was important that views be included from different treatment centers around the country. Where such evidence exists, purposive sampling to obtain diversity on these characteristics is indicated [28].

### 2.2 Data collection

#### 2.2.1 Semistructured interviews

Semistructured interviews following a predetermined interview schedule were conducted by the lead researcher (see Supplementary Material 2). This schedule was devised by members of the research team who provided clinical and methodological expertise. Following a review of the existing literature regarding qualitative studies in the field of DS and other conversion disorders, MW initially proposed topics to be included. These were added to/refined in an iterative manner by other members of the research team to ensure they covered a range of topics that might be relevant not only for characterizing the current therapists' experiences in the trial but also to highlight issues to be considered for a potential longer-term rollout of this intervention into different settings. The questions underwent revision to ensure that questions were appropriate and nonleading. Interviews lasted 40–60 min.

Where possible, interviews took place face-to-face at the clinicians' workplace. In three cases, geographical distance meant that the interviews were conducted via teleconferencing. Interviews were recorded on an encrypted digital recorder and were transcribed with identifying details removed. Anonymized transcripts were then analyzed using the NVivo v.12 (QSR International) software.

After completing the processes of familiarization and coding with the first 12 interviews, it was judged by the researchers that a point of data saturation had been reached: novel codes ceased to emerge from the data and many instances of existing codes were amassing [29]. Therefore, interviews were only conducted with these 12 therapists, all of whom gave written informed consent, and the remaining four identified therapists were not interviewed. Interviews were conducted between May 2017 and November 2017.

The process of qualitative data analysis also takes place within the context of power relations [30]. The interviewer (MW) was undertaking the interviews as part of a doctoral project at a separate academic institution from which the wider RCT was organized. Nevertheless, the interviewer may have been perceived as representing the wider project, and interviewees could, therefore, have felt under pressure to give comments that were affirming the value of the intervention. Moreover, through the process of keeping a reflexive research diary, the interviewer acknowledged that they could also experience a temptation to discourage negative narratives from emerging during the interview process, as this might be inconvenient for affiliated colleagues involved in the wider RCT. Furthermore, at times the interviewer could note in themselves a degree of defensiveness when interviewees voiced criticisms of the intervention. To try and mitigate such inclinations, the interviewer adhered as closely as possible to the interview schedule that had been devised to invite the open sharing of opinions (including criticism) and listened to previous interviews to be vigilant of such bias emerging.

### 2.3 Data analysis

Thematic framework analysis (TFA) was employed to analyze the interview transcripts. The originators of TFA sought to delineate a systematic process of qualitative analysis for research pertinent to the development of social and healthcare policy [31]. The approach is designed to be suitable for use in broader research projects that may employ multiple methodologies and large research teams [32,33]. Researchers have employed TFA in interview-based research that have explored the experiences of clinicians in different settings [[34], [35], [36]].

As prescribed by TFA, the qualitative analysis employed here consisted of five stages. Initially, two members of the research team went through a process of *familiarization* with the data, independently reading and rereading transcripts to develop initial thoughts regarding recurring ideas [32]. Following this, in a second stage (‘*coding*’), the researchers independently highlighted sections of text and applied paraphrases to indicate their interpretations. Other members of the research team then compared the two sets of coding to identify similarities and differences.

In a third methodological stage, a *theoretical framework* was devised where ideas from the coding stage were compared and grouped into common interpretative categories. While this initial theoretical framework emerged largely from the data, it was also informed by the a priori aims and theoretical background of the research [33].

In a fourth stage (‘*indexing*’) [31], the theoretical framework was applied back to the transcripts by the researchers to see how the raw data fit this framework. Adjustments to the framework were made iteratively as necessary. Once the indexing process was complete, the coded data were *charted*: excerpts of raw data were presented in a chart to illustrate a given category [31]. In a final stage (‘*mapping and interpretation*’) [33], the researchers considered the connections between the charted categories and explanations that might account for these connections.

## 3 Results

### 3.1 Participant characteristics

Sixteen therapists, identified through purposive sampling, were contacted via email to provide details of the project and ask whether they would like to participate. All contacted therapists gave email consent to be contacted to arrange an interview.

Table 1 provides demographic details of the participating clinicians. There was a good level of diversity regarding practice region, professional background, and experience delivering CBT interventions. All therapists were between 31 and 50 years old, and 10 were female. This distribution is consistent with the demographics of the wider CODES trial as well as those in psychotherapy professions, as seen by those applying to undertake clinical psychology training in the United Kingdom [37]. While the clinical services within which they were based differed in terms of the extent to which they would offer CBT or other psychotherapy to patients with DS outside of the trial, nine of the 12 therapists had prior experience of working with patients with DS.

**Table 1:** Participants' self-reported demographic information. Data are not presented by interviewee to preserve interviewees' anonymity.

Demographic characteristic	N	%
Age		
31–40	5	42%
41–50	7	58%
Gender		
Female	10	83%
Male	2	17%
Professional background		
Clinical Psychologist	4	33%
Counseling Psychologist	2	1%
Psychotherapist	2	17%
CBT Therapist	1	8%
Neurological Physiotherapist	1	8%
Nursing	1	8%
Occupational Therapist	1	8%
Highest level of CBT qualification		
MSc	2	17%
BSc	1	8%
Diploma	2	17%
No CBT-specific qualification	7	58%
Months of CBT training		
0–12 months	1	8%
13–24 months	4	33%
25–36 months	4	33%
37 +	1	8%
Data not provided	2	17%
Years practicing as CBT therapist		
0–5	6	50%
6–10	2	17%
11–15	2	17%
16–20	2	17%
Accredited with the British Association for Behavioral and Cognitive Psychotherapies	6	50%
Prior experience of working with DS		
Yes	9	75.0%
No	3	25.0%
Prior experience of working with medically unexplained symptoms		
Yes	10	83%
No	2	17%
Region		
Greater London	4	33%
North East England	3	25.0%
South East England	2	17%
South East Scotland	2	17%
Midlands	1	8%

The 12 participants had already treated or were treating a total of 108 CODES trial patients at the time of interview. The median number of patients per therapist already treated/being treated as part of the CODES trial was 8.5 (interquartile range: 3.25–12.75).

### 3.2 Themes

The process of mapping and interpretation yielded six overarching themes and 15 subthemes (see Table 2).

**Table 2:** Themes and subthemes.

Themes	Subthemes
1. Aspects of the intervention favored, while others were not always considered applicable	a. Seizure control techniques considered useful b. Family involvement considered useful c. Usefulness of graded exposure dependent on the presence and nature of avoidance d. Variable engagement with homework
2. Multiple and complex difficulties faced by patients	a. Comorbidities b. Therapist skill required
3. Working effectively within the protocol	a. Value of employing a structured approach b. Applicability to complex presentations c. Possibility of flexibility
4. Limitations of protocol	a. Sense of constraint b. Limitations of intervention c. Striking a balance
5. Significance of formulation	a. Standalone value of formulation b. Providing rationale for treatment and tailoring intervention
6. Quality of standardized medical care and difficulties of diagnosis delivery	a. Quality of standardized medical care b. Difficulties of diagnosis delivery

#### 3.2.1 Theme 1: aspects of the intervention favored, while others were not always considered applicable

##### 3.2.1.1 Seizure control techniques considered useful

The therapy manual outlined techniques for seizure control that most therapists reported to be useful when working with their patients. Interviewees reported that their patients typically experienced DS as uncontrollable, but that grounding or distraction techniques could introduce an increased sense of controllability that could alter their relationship with DS:

“When people are able to disengage, use the grounding techniques and the refocusing… they can see that they can engage in… thinking other than about the seizures and switch that pattern of behavior… that was a penny drop moment”

(Interview 4)

Two therapists stated that these seizure control techniques could help patients postpone a seizure but would not necessarily prevent their subsequent occurrence. The perceived usefulness of seizure delay was varied; one therapist stated that their patient consequently viewed their seizures as unavoidable and inevitable, though another interviewee reported that the ability to stall the seizure permitted engagement with valued activities:

“It can give them a sense of control, and obviously it could make a difference if, if they know that they are not going to have it in an embarrassing situation, that if they know, say if they are going out to, I don't know, a wedding”

(Interview 3)

Five participants remarked that the chapter in the book of handouts for patients that covered ‘Distraction and Refocusing’ techniques was especially useful, with two reporting that patients responded positively to the pragmatic and clear instructions. One therapist commented that having the chapter towards the beginning of the program allowed for the skills to be embedded early on and to be maintained independently.

Only two therapists commented on seizure occurrence during therapy sessions. One of these therapists indicated that seizure occurrence could be useful in developing seizure control techniques:

“it's never nice, ummm… but it's quite useful because then you can talk about it afterwards and you've got kind of a nice example right in front of you so you can kind of formulate it afterwards if they are able to sit with you and talk a bit about it.”

(Interview 2)

##### 3.2.1.2 Family involvement considered useful

In seven interviews, the therapists endorsed the emphasis on family work in this intervention, and there was an overall consensus that family was significant in recovery from DS. Participants suggested that the experience of a family member witnessing their relative's DS could be distressing and that this could lead to counterproductive protective behaviors:

“People kind of drop and they harm themselves, they may have knocked over a kettle and burnt themselves, so families have then become very protective and have maybe encouraged people to stop doing things from fear of them harming themselves.”

(Interview 5)

Participants viewed the inclusion of family members positively and appreciated the allotted treatment session as it allowed an explanation of treatment. One therapist stated that this was an opportunity to gather an observer's account of the seizures, while another commented on the potential for the session to enhance patient engagement by offering them control over which family members they would invite and when.

Three therapists described the new approach to managing DS as counterintuitive for family members. Being encouraged to step back from protecting their relatives during a seizure could be difficult to adjust to if they had prevailing concerns over a risk of harm. However, two therapists added observations about several families that welcomed the novel approach despite preexisting concerns:

“The urge to fall back into the old habits was quite strong… But on the positive side, I think that they were actually quite ready and maybe, you know, happy to, you know, use the information to take a little bit of a step back”

(Interview 11)

The chapter in the patient handbook aimed at family members was reported to be a particularly helpful tool by five therapists. Two therapists expressed surprise that the chapter had been shared with multiple family members and that this had enabled consistency in their approach to DS:

“I have given one to a family member, and even though they didn't speak English they seemed to kind of get it. And the family member was very supportive. Apparently then other family members read the booklet. And they were able to kind of follow the plan, and kind of learn about it”

(Interview 11)

Conversely, it is important to highlight that one therapist thought it notable not to overemphasize the family input in sustaining DS as there may be other systemic factors above the level of the family unit that may be relevant:

“there could have been a lot more scope to look at other things that might be perpetuating in the wider system… if you've got people in the family group acting in a certain way it can influence seizure behavior. That's one thread, but there seemed to be quite a big onus on that”

(Interview 10)

##### 3.2.1.3 Usefulness of graded exposure dependent on the presence and nature of avoidance

Consistent with the fear-avoidance model, the identification of avoidance behaviors and the subsequent decrease via graded exposure was foregrounded in this structured approach. Most participants reported some form of avoidance in their patients, and avoidance behaviors could be effectively treated through graded exposure. For example, when asked whether there were any 'light bulb moments' in treatment, one therapist gave the following response:

“Once they started to do some behavioral stuff and, and, and if they …went out and did something and found that their anxiety went down, that was a ‘light bulb moment’ for some people.” (N.b. 'light bulb moments' were interpreted to represent moments of realization or sudden understanding.)

(Interview 7)

Three therapists indicated possible shortcomings in the application of graded exposure in some of their patients, namely that avoidance behaviors were not always in evidence. Patients leading a normal work and social life did not necessarily report avoidance of specific situations, precluding the therapist from establishing graded exposure tasks. It was noted by six therapists that emotional avoidance could be present without overt behavioral avoidance. It was suggested that ‘exposure’ could be considered as exposure to avoided emotions:

“People were getting on with their lives as they normally would. I would say that the avoidance was really sort of an emotional avoidance. In which case being in the therapy was sort of facing that.”

(Interview 2)

##### 3.2.1.4 Variable engagement with homework

At the end of each treatment session, it was recommended that the therapist set homework tasks for the patient. Seven of the 12 therapists commented on the variable approach to, and completion of, homework. Different reasons were given for the noncompletion of homework that was seen as a general challenge within CBT. Such reasons included lack of time outside of sessions, comorbid difficulties, absence of behavioral avoidances around which to set homework, and literacy difficulties that led to embarrassment on the part of patients.

While it was clear that some therapists' patients had engaged well with the homework tasks and had benefitted, one therapist commented on a patient who had completed the homework tasks but had not improved. In addition, reference was made to other patients who did not complete the homework tasks and who maybe had not expected this to be part of therapy. It was deemed important to consider how it is best not to discourage patients where homework completion was not achieved:

“Yes… and also because I could see that s/he was not, s/he was not engaging with the, you know, with the homework. So s/he did, s/he would come back and say ‘oh I did try a bit of…’ and my, I guess my instinct as a therapist was to, you know, not to keep sort of maybe pushing or making him/her feel disheartened for something that s/he was ‘not achieving’, you know, like in inverted commas kind of thing, rather than — and instead to kind of focus on strengthening their belief in themselves.”

(Interview 11)

#### 3.2.2 Theme 2: multiple and complex difficulties faced by patients

Every therapist commented on the additional challenges faced by their patient group with DS. Comorbidities of mental and physical health issues increased the complexity of these cases.

##### 3.2.2.1 Comorbidities

All therapists reported witnessing a wide range of mental health difficulties including depression, obsessive–compulsive disorder, and emotional instability. The most prominent comorbidity reported by interviewees was that of trauma, although three therapists noted that not every patient experienced this. Therapists reported that, in two cases, the intervention was dominated by risk factors linked with the disclosure of trauma.

Other prominent features observed by several therapists were those of low self-esteem or social anxiety. This could have perpetuating effects on avoidance in day-to-day life.

Six therapists also commented on several of their patients facing severe physical health difficulties, for example, fibromyalgia, chronic pain, and varied cardiac issues. Although the therapists stated that they included the experience of these physical health issues in their patients' formulations, their occurrence may have affected the patients' anxiety levels during the exposure exercises and, in some cases could affect session attendance:

“…someone who already has avoidance and anxiety, and then they have physical health issues on top of that and don't feel physically well enough to come to sessions”

(Interview 5)

##### 3.2.2.2 Therapist skill required

Considering the comorbidities and complexities of the patients' experiences, three therapists suggested the necessity of suitably experienced clinicians to administer the intervention. Factors such as complex physical health difficulties and a history of trauma were used as examples in which a clinician's skill was important in guiding therapy:

“I think you have to understand the population. And I think you do need to have experience of complexity because you are not doing a cookie-cutter intervention at all.” (N.b. 'Cookie cutter' was interpreted here to represent something that was stereotyped or formulaic.)

(Interview 2)

#### 3.2.3 Theme 3: working effectively within the protocol

##### 3.2.3.1 Value of employing a structured approach

The majority of therapists reported that there were positive aspects of following the predetermined protocol. In some cases, the structure of the sessions was not considered applicable; however, the pacing and ordering of the prescribed intervention was reported to be appropriate for the patients. Four therapists stated that in cases where sessions may have drifted from the agreed aims, the protocol structure was effective at refocusing the sessions:

“People (meaning patients) are always inclined to take things into areas that they find are interesting to them, but I think what is quite useful about having a structure like this is that if you want to bring it back you can do it quite easily.”

(Interview 10)

##### 3.2.3.2 Applicability to complex presentations

Seven therapists shared the opinion that it was feasible to administer a structured protocol intervention to complex cases that included physical and mental comorbidities. One therapist commented on the efficacy of using the structure where individuals had an initial diagnosis of emotionally unstable personality disorder as the structure provided a means of containment. Overall, participants agreed that the structure gave clarity to the scope and capacity of the intervention:

“…they understood what the remit of the treatment was and how many sessions it was going to be… we had discussed what the treatment covered, but also what it hasn't been able to cover”

(Interview 3)

##### 3.2.3.3 Possibility of flexibility

Eight therapists reported having a degree of flexibility within the protocol. It was considered important to have space for reordering and placing specific emphasis on the features of the intervention when encountering the diversity and complexity of the patients. Some therapists reported greater confidence and trust in their own judgment following the supervision sessions that they received, reassuring them of the flexibility around their relationship with the manual. In three cases, interviewees suggested that they had an increased sense that they could make judicious use of the protocol components as they became more experienced.

#### 3.2.4 Theme 4: limitations of treatment protocol

##### 3.2.4.1 Sense of constraint

In the context of interviewing therapists who would not normally have their clinical work prescribed by working to an RCT protocol, five interviewees reported feeling constrained by the treatment protocol. Others said that they may have felt restricted had they not been allowed a reasonable amount of deviation rooted in clinical judgment about patient need. In some cases, therapists thought that the prescribed structure of the protocol led them to approach cases less ‘freely’ than how they had worked prior to the trial. Consequently, therapists felt that they could not integrate ideas and methods that they may otherwise have deemed relevant to patients' difficulties and may have had to act contrary to their usual inclinations:

“…the beliefs underpinning the model, being slightly different from my own, meant that it felt like I had a whole area of strategies and expertise and experience that I couldn't apply… I don't think it was a problem inherent in the model, I think it was the difference between how I normally work and how I was being asked to work”

(Interview 10)

##### 3.2.4.2 Limitations of the intervention

Some therapists reported a struggle in being able to work adequately with particular patients' needs within the protocol, and that they would have wanted to broaden the scope of therapy to address such issues. Notably, seven therapists experienced cases where they felt that patients were experiencing trauma-related issues that could not be addressed within the protocol, as this was not primarily a trauma-focused intervention. As a result, therapists reported a need for further referrals to trauma services after treatment had finished. Alternatively, in less complex cases, therapists felt that patients may have benefitted from a more limited intervention.

##### 3.2.4.3 Striking a balance

In three cases, interviewees indicated the need for a balance between rigidity and flexibility when delivering the intervention. These therapists suggested that unthinking adherence to the protocol could frustrate engagement and attunement to patient needs. However, the value of retaining a focus on the overall aims and structure of the intervention was also noted. It was suggested that the ability to strike this balance might come easier to therapists who had more experience to draw from:

“It was a balancing act. On the one hand we do want to stick to the model, we want to stick to the schedule of the treatment sessions… it's just using your discretion, using your experience I suppose. It's just not being too liberal with that. There are constraints”

(Interview 4)

Of relevance here also were therapists' reflections on the group supervision they received.

The importance of the supervision was acknowledged:

“Supervision is really important, yeh. I would say that the supervision was, has been kind of, not that the training wasn't good, it was good, but I would say that the supervision has been much more important in terms of maintaining fidelity to the model, answering questions and sort of trouble shooting difficulties.”

(Interview 2)

Not all therapists commented on the supervision that was available but, where comments were made, they tended to highlight the discussions that were held about the potential for flexibility within the protocol. Their comments highlighted the tensions that may have existed in achieving the correct balance for patients' treatment:

“So I think yeh, as I say some of the feedback from supervision has been that people have felt almost like compelled, that they've got to do it exactly as the manual says. And within that time they can feel a bit pressurized to fit that one particular session in”

(Interview 3)

However, supervision helped the therapists to be more aware of the formulation-driven approach being advocated in order to achieve the best approach for the patient in question:

“it's formulation-driven and the client, the patient is, you know, taking their best interests to heart, what they need in the context of the protocol is what I've understood to be the priority”

(Interview 6)

#### 3.2.5 Theme 5: significance of formulation

##### 3.2.5.1 Standalone value of formulation

Five therapists suggested that patients could benefit purely from the initial process of formulation, that is, the drawing together of information about what may have made the patient vulnerable to their disorder, what might have led to its emergence when it did, and what is maintaining it. It was reported that much of this value came from patients drawing links between past experiences and their current experience of DS and associating their thoughts and feelings with physical responses. Therapists reported that it could be helpful to understand potential relationships between multiple comorbidities that their patients may report at assessment. Developing this shared formulation could provide moments of profound clarity:

“…they kind of conceptualize the seizures in isolation from anxiety stress, early warning signs. So I think if you can kind of conceptualize it I think those are the kind of 'light bulb moments'

(Interview 1)

Four interviewees reported that their patients strongly identified with an account of their DS in terms of a reaction to unbearable emotion that precipitated a defensive, ‘switching off’ mechanism. This narrative could be sufficient to generate significant insight:

“The idea that it was too much and they were dissociating and that that may have been the reason for them starting. Most people could definitely relate to that”

(Interview 2)

##### 3.2.5.2 Providing rationale for treatment and tailoring intervention

Most therapists suggested that formulation was crucial to ground the intervention in the patients' experiences. By constructing a detailed formulation of an individual's difficulties and potential perpetuating factors, the therapists felt that patients had a clearer understanding of the rationale behind the interventions, and the therapists referred back to the formulation throughout treatment. One therapist advised that there was a risk of the model seeming ‘just academic’ without this individual contextualization. The formulation could also indicate the prioritization of some intervention components over others.

#### 3.2.6 Theme 6: quality of standardized medical care and difficulties of diagnosis delivery

##### 3.2.6.1 Quality of standardized medical care

Eleven out of 12 interviewees provided positive feedback on SMC that was provided as part of the RCT. Several therapists reported that their patients described positive experiences with neurologists or psychiatrists, feeling that they had taken the time to listen carefully to their concerns. Six therapists commented that they and their patients had benefitted from close working relationships with medical colleagues (i.e., neurologists and/or psychiatrists) during the trial. They suggested that this cohesiveness served to mitigate any sense that patients may have had of being abandoned by medical colleagues after being referred for psychological therapy:

“Because of the trial and because of the procedures in the trial, I was able to more easily work with the neurologist who had diagnosed the person with the condition, who was also reviewing them, and the psychiatrist as well which, felt a bit more like integrative and joined-up care”

(Interview 12)

Half of the therapists suggested that the quality of medical care within the trial compared favorably to that which patients would have received otherwise. Therapists reported that, outside of the trial, some patients could occasionally have negative experiences of the medical care received prior to therapy; this could make engagement in therapy more challenging. Therapists indicated that their patients within the trial were better informed and described aspects of the SMC approach that contributed to this. The booklets provided by neurologists and the standardization of the explanation given to patients [21] were mentioned as important factors:

“I think it's definitely helped that the neurologists have a… set sort of spiel to say to the patients, more of a standardized talk. And I know they have a leaflet that they give to the patients at that stage… it has definitely improved the level of knowledge”

(Interview 3)

Contrastingly, one therapist expressed a view that the CODES trial SMC was not necessarily superior to their usual care pathway, despite the fact that participants were typically receiving a higher level of input.

##### 3.2.6.2 Difficulties of diagnosis delivery

According to therapists, patients presented with varying degrees of understanding regarding their diagnosis and their reason for being referred to therapy. Five interviewees commented that patients attended their initial session with a good basis of understanding of the condition. They reported that their medical colleagues had delivered the diagnosis with clarity, as patients could give an accurate account of their condition. However, a similar proportion of therapists commented that there could be substantial limitations to their patients' understanding of the diagnosis and treatment. Typically, the therapists reported that their patients presented with a moderate degree of diagnostic understanding; this would include some sense that psychological factors were relevant to their difficulties:

“They would have some understanding, they wouldn't necessarily be able to join all the dots… but they will generally have a sense that it is a psychological model that is being proposed for their condition”

(Interview 4)

Where diagnostic understanding was poor, therapists did not automatically attribute this to a failure on the part of the referring neurologists and psychiatrists. Interviewees commented on the complexity of a DS diagnosis, and the difficulty of relating this in an intelligible way under time-restricted conditions:

“I think it's such a difficult concept for people to understand, so I don't think it's that they didn't hear it. I think they heard it and they tried their best, to try and get that over, but it's such a hard concept”

(Interview 5)

## 4 Discussion

Our aim was to explore therapists' experience and opinions of a structured DS-specific CBT intervention within the constraints of an RCT, where therapists were asked to follow a specific treatment protocol that may have differed from their normal practice. The study produced insights into three important areas: (a) strategies considered useful, (b) dealing with patient complexity, and (c) the structure and flexibility of the intervention.

### 4.1 Strategies considered useful

In addition to identifying the more general importance of formulation in the treatment process, techniques including grounding, distraction, and refocusing were considered particularly beneficial in helping patients to delay or prevent the occurrence of a seizure. Therapists felt that developing these skills empowered patients to regain a sense of control, which is especially relevant in DS, where patients characteristically lack a sense of power and control relating to their seizures [5].

Graded exposure was also highlighted as being an important factor in tackling avoidance and anxiety. However, therapists stressed that behavioral exposure exercises may not be appropriate for all patients, specifically those displaying emotional avoidance without overt behavioral avoidance. This presented a challenge to some therapists who felt that they could not apply the graded exposure section of the protocol to these cases. Psychiatrists within the trial also identified emotional avoidance as a specific challenge to treatment [38]. These concordant findings indicate the potential impact emotional avoidance may have on treatment outcomes and the importance of creating provisions and further guidance to tackle this within therapy. Of further therapeutic relevance are other observations of alexithymia [39] and altered emotional processing more widely in patients with DS [[40], [41], [42], [43]].

Another important component of the protocol was the emphasis on family involvement and psychoeducation. A significant number of therapists felt that engaging family members in the intervention by involving them in the treatment process and providing them with specific psychoeducation materials led to an increased support structure for patients. This has potentially important implications considering the percentage of patients with DS who experience communication challenges and issues with overprotective or overinvolved family members [[4], [5], [6], [7], [8], [9], [10], [11], [12], [13], [14], [15], [16], [17], [18], [19], [20], [21], [22], [23], [24], [25], [26], [27], [28], [29], [30], [31], [32], [33], [34], [35], [36], [37], [38], [39], [40], [41], [42], [43], [44], [45], [46]]. In this context, family therapy may be a valuable intervention for patients with DS. However, a systematic review [14] identified only one study using family therapy within DS treatment [47]. This study had patients' complete family therapy alongside other different interventions and was reported to be at high risk of bias. Future, more robust research examining the use of family therapy for DS would provide important insights.

### 4.2 Dealing with patient complexity

Although therapists generally felt that the protocol was applicable to most of their patients, the analysis revealed the importance of tailoring the intervention according to individual needs as patient presentation is heterogeneous. In cases where patients reported a variety of complications, formulation was considered paramount in order to link these difficulties, and this needs to be emphasized in future training of therapists treating patients with DS. Forming a coherent understanding of an individual case in this manner was often considered to be therapeutic in its own right.

Therapists felt that a level of experience and skill working with this patient group was necessary in order to carry this out successfully and adapt the intervention to specific cases (and in the CODES trial, supervision was also provided by experienced clinicians). This view was mirrored by the CODES psychiatrists who stressed the importance of clinicians having a significant level of experience working with DS to deal with challenging presentations, as well as being able to clearly explain the complexities of the condition to their patients [38]. This has implications also for the education and training about DS that needs to be provided to the broad range of healthcare professionals who may encounter and work with patients with DS [38].

### 4.3 Structure and flexibility of the intervention

The structure of the protocol was typically considered useful; however, it was clear that therapists felt that a degree of flexibility was necessary to engage patients and formulate an appropriate intervention. As indicated above with the case of graded exposure, the applicability of particular therapeutic strategies to an individual's needs had to be identified via a thorough formulation.

The SMC pathway designed for the trial was valued by therapists as it provided important structure and standardization to the treatment process. The elements of SMC highlighted as being particularly useful were the educational materials (booklets) and the standardized diagnostic explanation given to patients by both neurologists and psychiatrists. Notably, therapists generally indicated that their patients within the trial had a better understanding of their diagnosis than those they saw elsewhere. The importance of a clear and accurate diagnosis has been stressed by the neurologists and psychiatrists within this trial, emphasizing the detrimental affect diagnostic confusion can have on future treatment [23,38]. However, because of its complexity, not all patients were able to fully grasp their diagnosis, and some remained unclear throughout the treatment process; this will pose additional challenges for the therapists working with such patients with DS.

### 4.4 Limitations

Although the participants recruited into the study represented a significant proportion of all therapists involved in the RCT and was comparable with similar studies, the overall sample was small although saturation of themes was reached, and TFA does not prescribe sample sizes to be targeted [32]. Nevertheless, participants' experience was based on 108/186 (58%) of the patients allocated to receive CBT in the CODES trial, representing a substantial amount of trial experience. While we did not quantify the extent of prior experience working with patients with DS and could not investigate its influence on experience of delivering CODES CBT, further variability in responses may also have derived from differences in service arrangements in trial centers, which may have been associated with varying prior working relationships between the therapists and SMC doctors. Additionally, neurology/psychiatry services will have differed in their pre-CODES delivery of the diagnosis and patient management. However, the spread of settings in which the interviewed therapists worked represented the range of service arrangements across the trial more generally.

One potential significant limitation of this study is the lack of ethnic diversity among the patients treated by the interviewees. In the CODES trial, ~ 90% of participants identified themselves as white. The experiences of therapists in this trial therefore took place within a very specific cultural context, and there is evidence to suggest that individuals from other cultural backgrounds may have different understandings and experiences of functional neurological disorders such as DS. For example, Kendall and colleagues [48] reported that ‘hysterical conversion reaction’ patients in a Bangladeshi hospital would experience such symptoms in the context of culturally distinct stressors such as upcoming arranged marriages. It should also be noted that there is a general paucity of research into DS that employs nonmajority white, non-Anglophone samples. It is, therefore, important to acknowledge that this intervention may have been experienced very differently by individuals from different cultural backgrounds.

## 5 Conclusions

Analysis of interviews with CBT therapists identified important insights into the experience of delivering a structured intervention for DS; namely, challenges specific to the patient group as well as aspects of the protocol they found particularly useful or challenging. Analysis indicated that patients with DS often present with a complexity of mental and physical health problems in addition to their DS. The structure of the intervention often helped to guide therapists when dealing with complex cases; however, occasionally, they felt that additional psychological interventions may be required, particularly in cases with severe trauma.

Therapists found the application of treatment protocol components, such as seizure control techniques and family involvement, to be especially relevant. They also highlighted the importance of the formulation-driven approach of the intervention, whereby relevant treatment components could be prioritized according to the individual's needs. They indicated that a straightforward ‘one-size-fits-all’ approach should not be adopted, and this has implications for the experience of those delivering the intervention. Despite variable levels of diagnostic understanding reported at the start of therapy, there was a general consensus that the standardization of a care pathway, including neurological and psychiatric input, was beneficial for patients' understanding of their condition. Longer-term rollout of this intervention may need to address tensions inherent in asking therapists to adopt a different model of therapy for DS from that they are used to using.

The following are the supplementary data related to this article.

Supplementary Material 1 Topics covered in “Manual for Patients Attending CBT”. Supplementary Material 1

Supplementary Material 2 Interview schedule. Supplementary Material 2

## Declaration of competing interest

The authors have no conflicts of interest to declare.
