# Supplementary material for: The experiences of therapists providing cognitive behavioral therapy (CBT) for dissociative seizures in the CODES randomized controlled trial: A qualitative study
Source: Epilepsy Behav. 2020 Apr;105:106943. doi: 10.1016/j.yebeh.2020.106943 (PMC7156910; doi:10.1016/j.yebeh.2020.106943)
Supplement: Supplementary Material 1 — Topics covered in “Manual for Patients Attending CBT”. [file mmc1.docx]

**Supplementary Material 1**

**Topics covered in “Manual for Patients Attending CBT”**

Cognitive Behavior Therapy and Dissociative Seizures

A guide for other people

Distraction and re-focusing techniques

Progressive muscle relaxation exercises

Breathing exercises

Graded exposure

Trauma in the context of dissociative seizures

Identifying negative automatic thoughts

Alternatives to negative thoughts

Preparing for the future

Discharge plan
