# Supplementary material for: The experiences of therapists providing cognitive behavioral therapy (CBT) for dissociative seizures in the CODES randomized controlled trial: A qualitative study
Source: Epilepsy Behav. 2020 Apr;105:106943. doi: 10.1016/j.yebeh.2020.106943 (PMC7156910; doi:10.1016/j.yebeh.2020.106943)
Supplement: Supplementary Material 2 — Interview schedule. [file mmc2.docx]

**Supplementary Material 2**

**Interview schedule**

**I) What opinion do CBT therapists have regarding the efficacy, flexibility and design of this CBT intervention?**

1) How did you find the therapy manual and associated materials?

Possible sub-questions if needed:

a) How was it using the therapy manual/ the structure of the intervention on a sessional basis?

b) What did you think about the ordering of the sessions/ Was any re-ordering necessary

c) Which aspects of the intervention did you feel were easier/ more difficult to deliver?

d) Did you tend to direct clients to particular readings in the booklet for clients?/ Were any of the chapters in the clients’ booklet more/less useful?

2) How was it working within the CBT protocol? What would you say about the flexibility of this approach? (or was that what you meant by the question I added in above?)

3) If this CBT intervention were to be rolled out across other services, what changes, if any, would you make to it?

4) If you were not working under the constraints of the trial, would you have applied a different therapeutic model, and if so what? Did that cause any tension for you?

**II) What experience do CBT therapists believe their clients had of the intervention? What experience did CBT therapists have of delivering the intervention?**

1) What would you say about your clients’ ability to relate the CBT model to their difficulties? How satisfying/meaningful did clients tend to find this as an explanation for their problem?

2) Were there any ‘lightbulb moments’ in the course of treatment where clients appeared to have a sudden understanding of their treatment? (Prompt if needed: If so, at what point in treatment did this occur/could you describe the nature of this moment?)

3) Did the way clients engaged with therapy seem to change over time? (Prompt if needed: Could you say something about the nature of this change)

4) Did your experience of providing this intervention change over the course of the trial? (Prompt if needed: If so, in what ways did this change?)

**III) What psychological processes did CBT therapists think that they were targeting in the intervention? Did therapists perceive individual psychological, social or health-related differences between clients that made it easier or more difficult for them to benefit from the CBT intervention?**

1) What psychological processes did you think that you were targeting (directly or indirectly) in the intervention?

Possible sub-questions:

a) Did fear-avoidance feature in your clients’ presentations?

b) If trauma was a significant feature of your client’s presentation, how did you approach it in the context of this intervention?

2) Were there characteristics of clients that made it easier or harder for them to work with the treatment? (Prompt: what were these characteristics? How did they affect the course of treatment? If we think about a particular client…)

3) Were there issues that you had to address in order to improve engagement? (Prompt: could you any examples of this? Were there any issues regarding timing/location/travel/childcare/need for relative support?)

4) Could sessions ever become side-tracked/derailed by other issues? e.g.: social issues, safeguarding or health-related concerns (Prompt: could you give any examples of this? How easy was it to come back to the focus of treatment?)

**IV) How did CBT therapists experience the overall care pathway, and how well integrated, in their opinion, were the CBT and SMC aspects of treatment?**

1) What did you think about the overall care pathway? (Prompt: How did SMC sit alongside the CBT intervention/ What would you say about the integration of these two aspects of treatment?)

2) Did clients discuss their experiences of SMC in CBT sessions, and if so what did they report? In what ways did this seem to influence their understanding of their condition?

3) Do you feel that your clients understood their diagnosis? What do you think this diagnosis meant for them?
